# Approved and investigational fluorescent optical imaging agents for disease detection in surgery

**DOI:** 10.1097/JS9.0000000000000459

**Published:** 2023-05-16

**Authors:** Sonia Rehman, Paul M. Brennan, Annamaria Lilienkampf, Mark Bradley

**Affiliations:** a School of Chemistry; b Centre for Clinical Brain Sciences, The University of Edinburgh, Edinburgh, UK

**Keywords:** fluorescence, intraoperative, optical medical imaging, peptides, real-time imaging, surgical

## Abstract

Fluorescent optical imaging is becoming an increasingly attractive imaging tool that physicians can utilise as it can detect previously ‘unseen’ changes in tissue at a cellular level that are consistent with disease. This is possible using a range of fluorescently labelled imaging agents that, once excited by specific wavelengths of light, can illuminate damaged and diseased tissues. For surgeons, such agents can permit dynamic, intraoperative imaging providing a real-time guide as they resect diseased tissue.

## Introduction

Highlights

- Optical medical imaging is fast becoming a useful tool within the clinical setting.
- Optical imaging agents allow for disease visualisation and monitoring based on molecular signatures.
- Fluorescently targeted imaging agents can provide surgeons within additional intraoperative guidance during the resection of diseased tissue.
- This review gives an overview of fluorescent imaging agents currently utilised and in clinical trials.

Medicine evolved rapidly during the 20^th^ century, with new procedures, revolutionary drugs, and technologies. Among these are a suite of medical imaging techniques and procedures. Medical imaging relies on the old adage ‘seeing is believing’, except here the ‘belief’ is really a confirmation that there is a need for treatment that may range from an administration of a drug to a surgical intervention, or indeed both, since current imaging scenarios do not lead to direct intervention in their own right. Current clinical imaging methods include MRI, positron emission tomography and computed tomography/X-ray, which are used both preintervention and postintervention, but from a clinical standpoint there is a huge unmet need as there is often little to monitor and to ‘see’ what is actually happening during the intervention. Now in the 21^st^ century, opportunities such as optical medical imaging have begun to emerge that synergise the molecular basis of disease with fluorescent technologies. These provide imaging based on biomarkers that are disease dependant such that treatment and disease progression can be monitored at a cellular/molecular level. In addition, as optical molecular imaging techniques utilise the visible light spectrum, these imaging techniques not only reduce the need for exposure to radiation but also offer exquisite molecular sensitivity.

## Optical medical imaging

Optical imaging encompasses a suite of imaging tools to visualise and indeed interrogate structures, cells and tissues through the use of the visible and deep red spectrum of light (400–900 nm). Examples of clinically relevant optical imaging modalities include endoscopy, optical coherence tomography, photoacoustic imaging, and fluorescence^1^. X-ray and positron emission tomography are also optical imaging techniques, but this review will use the term ‘optical imaging’ in relation to the more ‘classical’ visible spectrum of light.

Optical imaging can allow the visualisation of disease at a molecular level and has begun to use a range of fluorescent techniques as a powerful tool for, not only disease diagnosis, but also for following disease progression and treatment monitoring, for example, imaging disease dependant biomarkers. One particular area of interest in a clinical setting is fluorescence guided surgery, allowing surgeons to monitor and investigate molecular signatures consistent with disease as they operate in real-time^2^. It is beneficial for patients and surgeons alike if fluorescence guided surgery can aid in delineating the margins of disease that can often be invisible/unclear and results in poor surgical margins, that is, surgeons under-cutting and leaving diseased tissue behind, the need for repeated surgery, or the removal of healthy tissue and disfigurement.

Fluorescence imaging uses light emitted following excitation of a fluorescent compound. In the context of optical-based imaging within a clinical setting, fluorophores with emissions above 650 nm are more conducive to *in vivo* imaging as this avoids the background fluorescence or ‘noise’ that results from naturally occurring endogenous fluorophores such as porphyrins, tryptophan, reduced nicotinamides, flavins, lipo-pigments and folate^3^. This review introduces fluorescent probes that are currently approved for use in the clinic or are in clinical trials and under investigation for fluorescence guided cancer surgery.

## 5-Aminolevulinic Acid (5-ALA)

**Figure 1:**
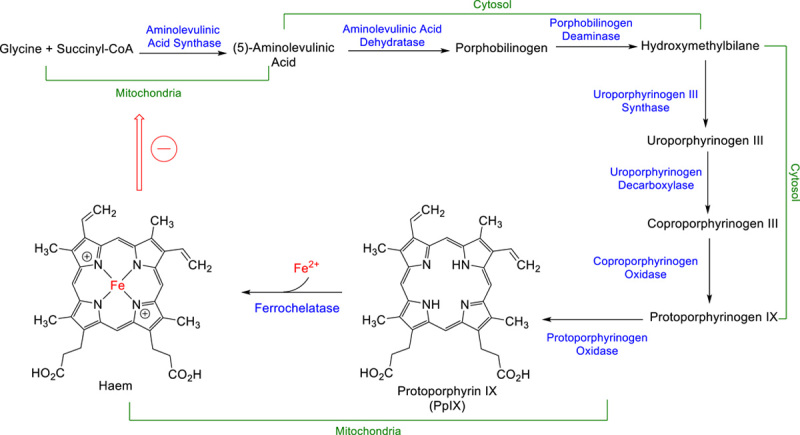
Overview of the haemoglobin biosynthesis pathway, showing the major metabolic steps and enzymes (in blue) to form haem prior to its conjugation to a protein to form haemoglobin. In healthy tissue, haem formation has a negative feedback effect on aminolevulinic acid synthase, allowing the cell concentrations of 5-ALA and, by extension, haem to be controlled^5,6^. In cancerous tissue, reduced ferrochelatase expression level results in a build-up of the PpIX intermediate that is fluorescent (emits at 635 nm when excited at 405 nm).

5-Aminolevulininc acid (5-ALA) is used in glioma resection by neurosurgeons. 5-ALA is an endogenous metabolite generated from succinyl-CoA and glycine and is an intermediate along the haemoglobin biosynthesis pathway^4^. The biosynthesis pathway generates haem prior to the formation of haemoglobin (Fig. 1).

Along this pathway, fluorescent protoporphyrin IX (PpIX) (emits at 635 nm when excited at 405 nm) is formed in the step before haem formation. Within healthy cells, PpIX is metabolised by ferrochelatase to haem. Within glioma cells, there is a reduced level of ferrochelatase expression, resulting in both a build-up of fluorescent PpIX and a lack of negative feedback, which results in the continual fuelling of 5-ALA into the haemoglobin biosynthesis pathway^7^. Additionally, the haemoglobin biosynthetic pathway is upregulated in high-grade gliomas due to cells undergoing uncontrolled proliferation^8^.

**Figure 2:**
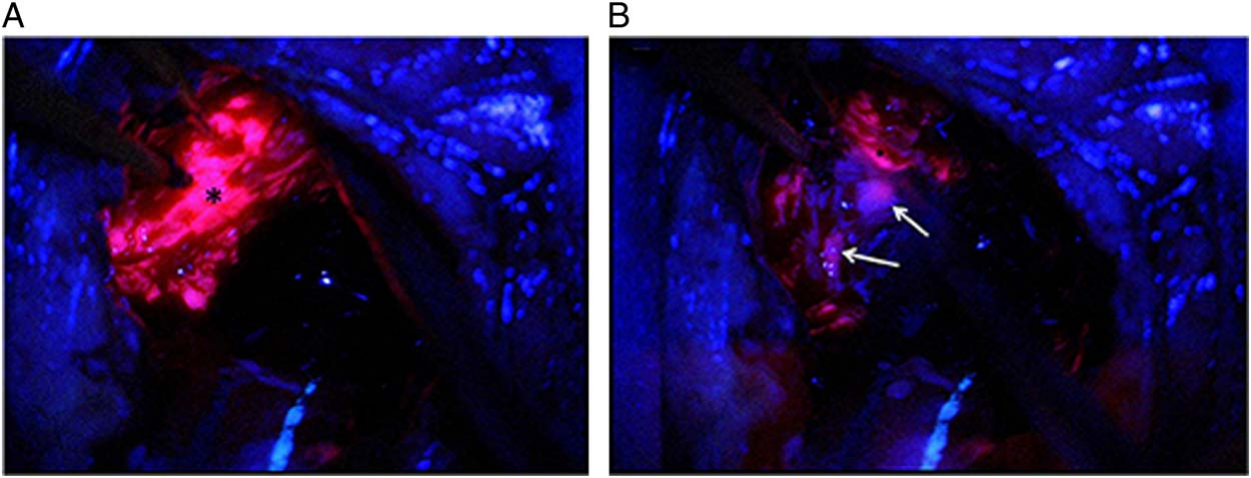
Example of the fluorescence imaging capabilities of 5-ALA (administered as 5-ALA hydrochloride) in visualising a high-grade glioma. 5-ALA imaging helps tumour identification (in pink) and delineation of surrounding healthy tissue (in blue). Areas of dense, necrotic tissue in the tumour core that are no longer metabolising are unable to generate/accumulate the fluorescent PpIX. Proliferating solid tumour (in A annotated by*) and regions of diffuse tumour infiltration (in B indicated by white arrows) where margins are unclear are demonstrated in the image above^11^ (left). Image reproduced from open access article^11^ with permission from Frontiers Media.

5-ALA was approved in 2017 by the FDA for the visualisation and removal of high-grade gliomas (high grade glioma, WHO Grade III or IV) in adults^9^, although it was in widespread use by neurosurgeons for many years prior to its approval. 5-ALA, when administered, is effective in ‘optically highlighting’ more than 90% of high grade glioma^10^. An example of the fluorescence emission from a tumour following PpIX accumulation from 5-ALA administration can be seen in Figure 2. Interestingly, a systemic review that collated results from multiple studies found that 5-ALA administration resulted in fluorescence in less than 25% of low-grade gliomas^12^. Commercially available 5-ALA (formulated at 5-aminolevulinic acid hydrochloride) is available for use with a recommended dose of 20 mg per kg of body weight^13^.

More recently, 5-ALA has been used for photodynamic therapy (PDT) as a treatment option for cancers. PDT uses photosensitisers such as porphyrins, which, upon excitation, become activated and generate singlet oxygen (among other reactive oxygen species) in the proximity of cells. This drives apoptosis and cell death^14,15^. 5-ALA has been investigated as a PDT treatment option for cancers including malignant glioblastomas, superficial bladder cancer, and other neoplasias^16^. A current Phase II clinical trial is investigating 5-ALA as a form of PDT for benign dermal neurofibromas as well as other superficial skin cancers^17^.

### EMI-137

c-MET (a Hepatocyte Growth Factor Receptor) binds the Hepatocyte Growth Factor (HGF), a plasminogen-like protein. Once bound to c-MET, HGF mediates cellular repair and regeneration in healthy tissue^18,19^. The c-Met receptor is part of the tyrosine kinase family of receptors from the MET gene family and c-MET receptors have been implicated in a number of cellular pathways including cell signalling, proliferation, growth, development, migration and invasion within the context of cancer^20^. Overexpression of the c-MET receptor has been found in the tumour growth and development stages (specifically angiogenesis, cell invasion and metastasis stages of tumour growth) of cancers and other diseases including glioblastoma, Barrett’s oesophagus, colorectal cancer and colitis^21,22^.

**Figure 3:**
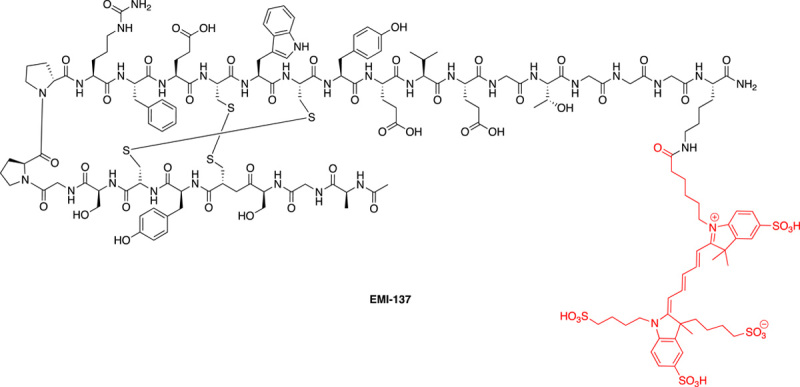
Chemical structure of EMI-137 showing the disulphide bridges between cysteine residues 4-16 and 6-14. The Cy5** fluorophore (in red), bearing four sulphonate groups to promote solubility, is conjugated to the targeting peptide via a small peptide spacer (GGGK).

EMI-137 (also called GE-137) is a Cy5 fluorophore labelled multi-cyclic peptide that binds c-Met, which is overexpressed in cell membranes within diseased colon tissue. This peptide ligand was discovered from phage display/selection where several peptides were identified that had different sequences and modes of function. Thus, some peptides showed binding to c-MET and inhibited HGF binding, other peptides were shown to bind at another site on the c-MET receptor, allosterically inhibiting HGF binding^23^. The peptide AGSCYCSGPPRFECWCYETEGT (containing two intramolecular disulphide bridges) showed good binding affinity to the c-MET receptor (K_d_ = 3 nm) and was found to bind to a site on the receptor that did not impinge on HGF binding and was thus selected for further studies^23^. EMI-137 is composed of a *bis*-disulphide 22-mer c-MET targeting peptide, with a C-terminal primary amide and an acetylated amino terminus, while a GGGK spacer allows conjugation of the near infrared (NIR) Cy5** fluorophore (Ac-AGSCYCSGPPRFECWCYETEGT-GGGK(Cy5**)-NH_2_)^24^ and separation of the dye from the targeting peptide (Fig. 3). The Cy5** fluorophore used gives the probe excitation and emission wavelengths of 650 nm and 669 nm (the Cy5** nomenclature depicts its multiple sulfonations that promotes its solubility).

**Figure 4:**
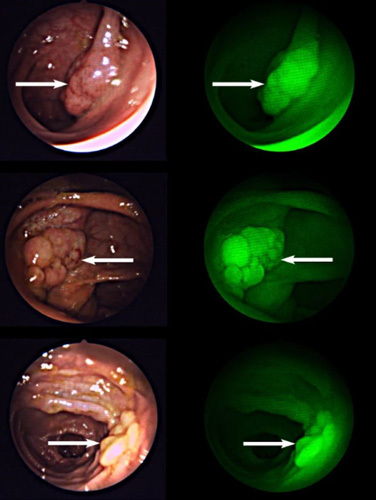
Images from colonoscopy of bowel colorectal polyps when the patient has been administered with EMI-137. Images on the left show the comparison of visualisation using standard white light (in the field of view in an endoscopy/colonoscopy) compared to the fluorescent images (in green) showing ‘unseen’ polyps that were not as evident in white light from the application of EMI-137^24^. Image reproduced from article^24^ with copyright permission granted from Springer Nature.

EMI-137 has been investigated to image colorectal polyps that are found to be a prominent pathological feature of colorectal cancer, colitis and Crohn’s disease^25^. The inflammatory nature of colorectal polyps makes visualisation by colonoscopy or exploratory surgery difficult as polyps are often surrounded by inflamed bowel tissue that can disguise diseased tissue. Intravenous administration of EMI-137 target overexpressed c-MET receptors in the polyps allowing visualisation of polyps when excited at 650 nm (using a custom designed/built system) during colonoscopy. The identification of bowel polyps using fluorescent EMI-137 compared to standard white light colonoscopy can be seen in Figure 4.

**Figure 5:**
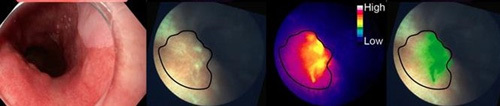
Comparison of traditional ‘white light’ imaging and fluorescence imaging (excitation at 650 nm) with an overlay of images of the inflamed oesophagus when EMI-137 was administered IV. The overlay highlights (in green) where EMI-137 has concentrated likely where there is increased c-MET receptor expression^27^. Image reproduced from open access article^27^ with permission from Ivyspring International.

EMI-137 is currently in Phase IIb clinical trials for the identification, detection and screening of colorectal polyps and cancers. Studies include the investigation of whether EMI-137 is capable of producing fluorescence and detecting primary tumours (that are confirmed to be c-MET positive by histology) whilst developing an *in vivo* safety profile, with studies also exploring whether EMI-137 can detect cancer draining lymph nodes where metastasis has been confirmed by histological studies^26^. EMI-137 has also been investigated in a Phase I trial for the detection of Barrett’s oesophagus – a precancerous disease. When compared to standard white light, EMI-137 localisation to lesions was found to provide a gradient of visualisation of expressed c-MET receptors (Fig. 5) that could allow targeted treatment within the area of dense fluorescence. In this investigation, EMI-137 was administered intravenously at up to 0.13 mg per kg of body weight^26^.

### OTL38

**Figure 6:**
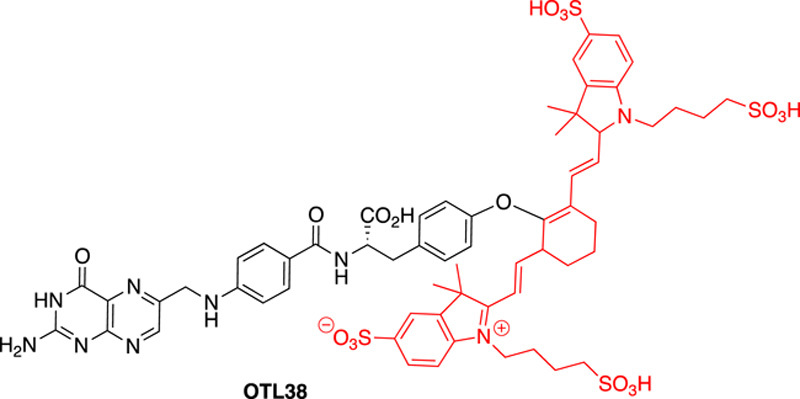
Chemical structure of probe OTL38 where a folate-type ligand is conjugated to the SO456 cyanine-like NIR dye (in red) via a tyrosine residue.

OTL38 is a NIR imaging probe based on folic acid coupled to an indole cyanine-like green dye (SO456) (Fig. 6). In synthesising this probe, the glutamic acid residue of folate was replaced with a tyrosine that was *O*-alkylated with the SO456 dye^28,29^. The replacement of the glutamate by tyrosine gives an increase in fluorescence intensity by more than 200% (when compared to the SO456 dye alone). It is thought that the conjugation of the tyrosine to SO456 contributed to electron delocalisation and brought about the noted fluorescence intensity^29^. OTL38 is excited at 776 nm and emits at 793 nm.

**Figure 7:**
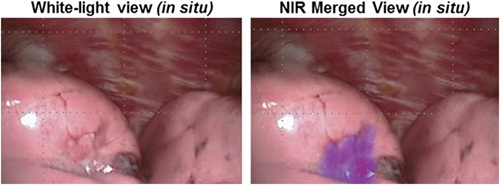
Comparison of in situ tumour visualisation of pulmonary squamous cell carcinomas in white light alone (left) and merge image with OTL38 fluorescence at 776 nm. Overlaying the OTL38 signal identifies FRα+ve tumour tissue to allow for focused resection of diseased tissue^39^. Image reproduced from open access article^39^ with permission from Elsevier.

OTL38 targets the folate receptor alpha (FRα) overexpressed in several cancers. More than 80% of ovarian tumours^30^ and 74% of pulmonary adenocarcinomas^31^ have increased FRα expression^32^. FRα, expressed by the FOLR1 gene, is typically activated and expressed in early embryonic development^33^. Thus, in the context of healthy tissue, there is low FRα expression but increased expression is observed in cancerous tissue^34^. Activation of FRα is thought to be involved in episodes of rapid cellular proliferation with folate binding supporting DNA synthesis prior to mitotic division^33,35^. With high levels in diseased tissue and low levels in healthy tissue, FRα is an attractive biomarker for imaging ovarian and lung cancer. OTL38 was approved by the FDA in 2021 as Pafolacianine after its Phase III clinical trials for the intraoperative identification, imaging and resection of FRα positive ovarian and lung cancers provided promising clinical data^36,37^. A dose guidance of up to 0.025 mg per kg of body weight was advised administered as a continuous intravenous infusion over 60 min up to 9 h before surgery^38^. An example of the imaging capabilities of OTL38 from Phase II studies are shown in Figure 7.

### BLZ-100

BLZ-100 (one of the compounds in the Tumour Paint series by Blaze Bioscience) has potential for use in surgical resections of solid mass tumours^40,41^. BLZ-100 consists of a chlorotoxin (CTX) peptide to which the fluorophore indocyanine green (ICG) has been attached via NHS ester formation – this results in a NIR probe with an excitation/emission of 675 nm/740 nm, whilst ICG alone has an excitation/emission of 789 nm/814 nm (see section on ICG). The use of ICG as a NIR fluorescent dye in BLZ-100 allows for an ease of accessibility in using this probe as its emission window can be imaged using existing and currently available optical imaging hardware currently used in clinical environments. BLZ-100, when intravenously administered was found to be safe for use at a dosage of up to 30 mg per kg of body weight in Phase I safety and pharmacokinetic investigations^41^. Currently, BLZ-100 is in several Phase I clinical trials for use in skin, brain and breast tumours, and in Phase II and III clinical studies for paediatric central nervous system tumours^42^. BLZ-100 has been fast tracked by the FDA for paediatric central nervous system tumours.

**Figure 8:**
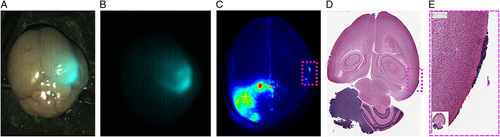
(A, B) injected BLZ-100 and a whole mouse brain imaged using a clinical prototype imaging ‘SIRIS’ system with the tumour highlighted; (C) whole mouse. brain imaging of IV BLZ-100 with peripheral tumour cells highlighted; (D, E) histological staining showing tumour tissue^46^. Image reproduced from open access article^46^ with permission from Cureus.

Chlorotoxin (CTX) is 36 amino acid peptide (MCMPCFTTDHQMARKCDDCCGGKGRGKCYGPQCLCR) with eight cysteines forming four disulphide bonds making it structurally very compact and protease resistant^43,44^. It was initially isolated from the scorpion venom of *Leiurus quinquestriatus* and binds to chloride ion channels. Chloride ion channels are overexpressed in cancers such as glioblastoma, lung and skin cancer. In the context of cancer, chloride ion channel overactivity can result in a regulatory volume decrease in cells that can be brought about due to hypotonic stress^45^. Regulatory volume decrease contributes to phenotypic changes seen in cancer development at a cellular level and can cause changes in cell shape and increase cellular migration^45^. Chloride ions and channels also act as co-transporters for potassium ions whose influx in known to be implicated in tumorigenesis. CTX’s tumour binding capabilities were first investigated in rat glioblastoma models were CTX was found to bind to gliomas, but interestingly did not bind to healthy rat astrocytes^43^. CTX has therefore become an attractive targeting ligand in tumour binding studies. The focus of BLZ-100’s glioblastoma identification and delineation in mice brains showed fluorescence at probe concentrations as low as 500 pM with BLZ-100 showing no binding or fluorescence in healthy mice brain tissue (containing a cross section of neuronal and astrocyte cells (Fig. 8)^46^.

### LUM015

Cathepsins are a class of cysteine proteases with a wide range of activities and are specifically noted for their involvement in remodelling type II, IX and XI collagens and proteoglycans. Their activity is regulated by cytokine signalling and Ras proto-oncogene expression, with mutations in the Ras proto-oncogene associated with 70% of colorectal cancers and 47% of carcinomas^47^. The overexpression of multiple types of cathepsins has been linked to the development and progression of many cancers, with overexpression enabling cancer progression by aiding cell proliferation, tissue modelling and angiogenesis, tumour growth and cellular metastasis^48^. Cathepsins B in particular has been associated with invasive and metastatic phenotypes typically found in aggressive forms of cancer.

**Figure 9:**
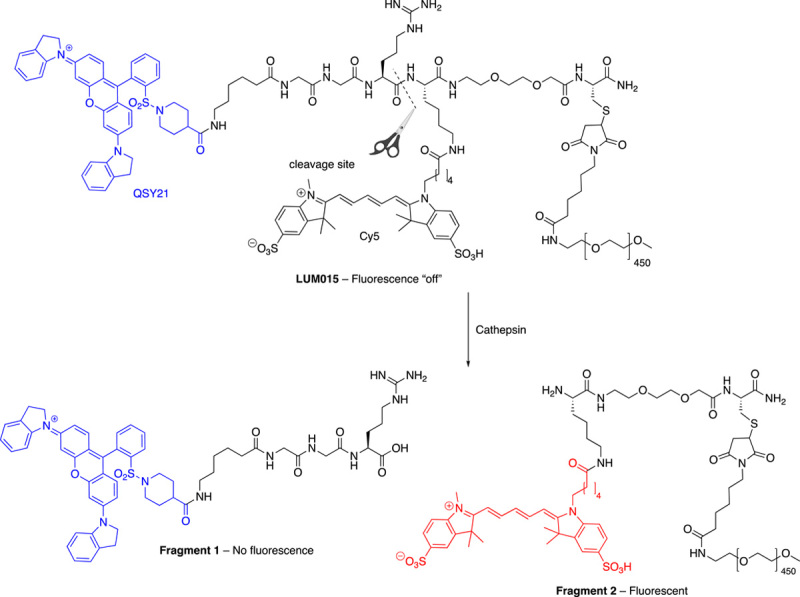
The structure of the FRET-based LUM015 and the fragments resulting from cathepsin cleavage at the Arg–Lys amide bond. Fragment 1 contains the QSY21-GGR. Fragment 2 is composed of the fluorescent Cy5 dye attached to the 40kD PEG chain via the lysine residue. Another fragment found is Lys-Cy5 where the PEG chain has been cleaved. Fragment 1 is optically inactive when excited at 649 nm, whilst fragment 2 emits at 670 nm.

LUM015 has been described as an ‘onco-fluorescent’ and protease-cleavable deep-red probe^49^, whose fluorescence is quenched until cleaved/activated. It consists of the quencher QSY21 attached to the N-terminus of a small peptide (sequence GGRK), with the C terminus linked to a 20kD PEG unit. QSY21 acts as a quencher for the Cy5 fluorophore (FRET pair) attached to the lysine side chain (QSY21-GGRK(Cy5)-PEG-OMe)^50,51^. The addition of the PEG unit promotes tumour accumulation when administered intravenously^52^. This probe was designed to target the microenvironment around tumours and becomes optically active in the present of cathepsins and matrix remodelling^49^ with cleavage of the Arg–Lys bond by cathepsins K, L, S and B (Fig. 9) liberating the Cy5 fluorophore (excitation and emission wavelengths are 649 nm and 670 nm). LUM015 is currently in Phase III clinical trials for intraoperative use in detecting residual breast cancer tissue is patients undergoing standard lumpectomy^53^. This study administered LUM015 to patients intravenously up to 6 h prior to lumpectomies at a dose of 1.0 mg per kg of body mass. Imaging of tumour masses was carried out using the Lum System for intraoperative imaging and guidance^53^.

### Indocyanine green (ICG)

ICG, as the name suggests, is a tricarbocyanine dye available in both sulfonated and nonsulfonated forms. The sulfonated dye is used clinically as it is freely soluble in water giving rise to a green solution that fluoresces in the NIR-I window (700–900 nm) with an excitation and emission maxima of 784 nm and 814 nm, respectively (although these values are subject to small changes dependant on the dye’s environment).

Early recorded use of ICG date back to the late 1950s, where it was used in the study of cardiac blood flow^54^. Investigations found that once administered, the dye was both rapidly distributed and cleared with more than 97% of the loading dose demonstrating hepatic excretion via the bile duct with little to no alteration of the dye’s structure^55^. In its native form, ICG is untargeted (unlike, for example, BLZ-100) and shows low toxicity (LD_50_= 80 mg/kg) – a property that stems from its lack of binding and rapid clearance with a half-life of only 4 min^56^.

**Figure 10:**
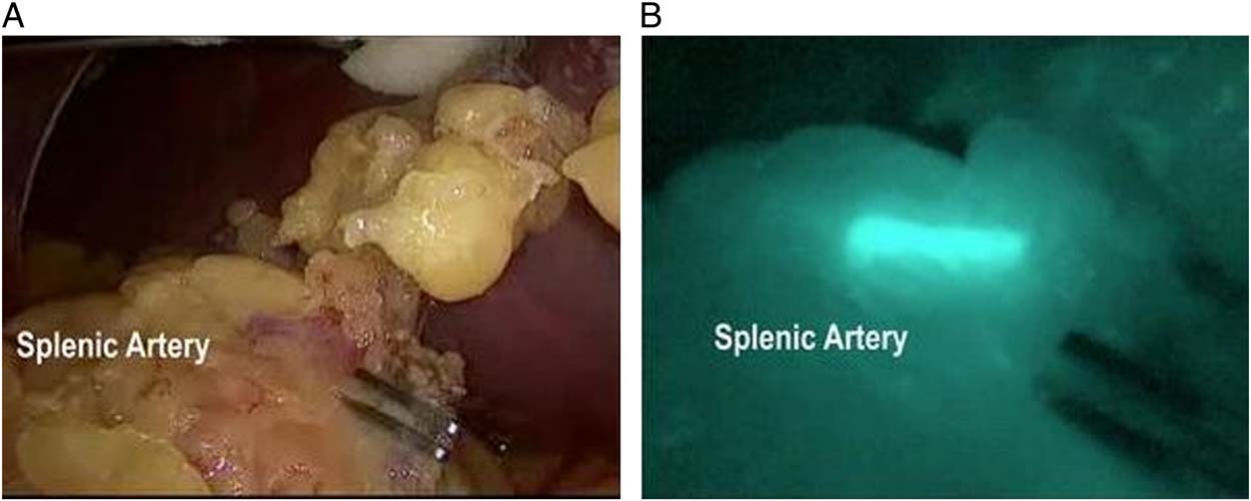
Comparison of the splenic artery provided by standard white light (left) and ICG fluorescence (right). ICG diluted in saline was administered intravenously and imaged using the ICG-Pulsion by pulsion medical system. In the presence of ICG, visualisation at 800 nm provides a more readily defined splenic artery outline than white light alone. Image reproduced from open access article^58^ with permission from Springer Nature.

Use of intravenously administered ICG includes angiography, where the dye provides blood vessel contrast that may be subject to occlusion, damage or trauma and for assessing intraoperative myocardial perfusion^57^, with an example of the imaging capability of ICG in viewing vascular anatomy shown in Figure 10 ^58^. Typical dosages required for angiography can range from 0.1 to 5.0 mg/kg and are highly dependent on the clinical investigation^59^.

Importantly, the emission window of ICG also extends into the near-infrared-II window (NIR-II 1000 nm–1700 nm). This has proven to be clinically important as NIR-II light undergoes reduced scattering with tissue (1/λ^4^) and with lower levels of autofluorescence than in the visible or NIR-I region. In addition, studies have demonstrated that with a disrupted blood–brain–barrier ICG can accumulate in GBM^60^. Recent examples of ICG with visualisation in the NIR-II window have yielded promising results. In a first-in-human investigation assessing tumour detection using a multi-spectral imaging system, it was found that when administering ICG in patients with confirmed metastatic liver tumour, NIR-II had a higher tumour detection sensitivity rate than NIR-I imaging (100% compared to 91%, respectively)^61^. A similar finding was reported by Shi *et al*.^62^ who compared the effectiveness of high-grade glioma detection and resection between NIR-II (using ICG) and conventional white light surgical guidance. Here, it was demonstrated that NIR-II ICG fluorescence guided surgery had a higher accuracy rate in detecting tumour tissue than white light detection (97– 81%). This comparison was carried out using data from intraoperative guidance as well as ex-vivo imaging to compare resected tumour tissue to surrounding healthy tissue margins^62^. The use of ICG within the context of the brain is particularity interesting as it could be useful in the investigation of not only tumour tissue, but also potentially of blood flow in microvasculature given its ability to bypass a disrupted blood–brain–barrier.

### The future of optical imaging in surgery

Fluorescent optical medical imaging is a powerful technology just beginning to show glints of its full potential within the clinical arena – Table 1 provides a brief overview of the imaging agents discussed within this review. There are many other imaging probes under investigation yet to enter human studies and surgeons will be called upon to develop the use cases and evidence of clinical validity. We have highlighted applications in cancer but there will be broader application in surgery, infection monitoring and disease diagnosis and monitoring as well as cancer margin delineation—all areas that are under active investigation. As an example, optical probe NP41, a peptide based reagent composed of a Cy5 fluorophore coupled to a nerve targeting peptide^63^ highlights myelin encased nerves. NP41 could be help identify nerves intraoperatively for preservation^64,65^. A range of fluorescent molecules have been designed for imaging fungi (e.g. fluorescent Amphoterocin B-based probes^66^) or bacteria which could aid in the removal of infected tissue. As imaging technologies and detectors become ever more sophisticated, fluorescent imaging that moves beyond the traditional optical window has advantages. For example, there is reduced light scattering by tissue at longer wavelengths and in this review dyes operating beyond 1000 nm show much promise. In addition, the value of newer imaging modalities, such as fluorescent lifetime, in which the long-known, intrinsic, fluorescent lifetime variation between healthy and diseased tissue offers huge promise in cancer margin determination, perhaps linked to other optical methods such as Raman. Optical medical imaging is a powerful technology and its application will support surgeons and robotic surgery in the diagnosis, localisation and treatment of disease.

**Table 1:** Summary of imaging agents discussed in the review (both FDA approved and investigational new drugs) highlighting the disease type, area targeted by probe, structure-type, approval status, optical properties, and reported dosage.

Imaging agent	Disease	Target	Structure-type	Approval status	Optical properties (wavelength of excitation/emission)	Reported dose
5-ALA^4,10,17^	High-grade glioma imaging and resection.	Haem biosynthesis pathway.	Small molecule that is incorporated into the haem biosynthesis pathway.	FDA approved for use in high-grade gliomas.	405/635 nm	20 mg per kg (administered as Gliolan).
EMI-137^25,26^	For use in imaging in colorectal, breast, head and neck and lung cancer.	Overexpressed c-MET receptors.	22 mer peptide labelled with Cy5**.	Currently in Phase IIb clinical trials for colorectal cancer and Phase I for breast and head and neck.	650/669 nm	0.13 mg per kg
OTL38^28,30,38^	Indicated for use in imaging of ovarian cancer for surgical resection.	Targets overexpressed FRα present in tumour tissue.	Small molecule targeting FRα coupled to a NIR dye.	Gained FDA approval for use in imaging of ovarian cancer (Pafolacianine) in 2021.	776/793 nm	0.025 mg per kg (administered as Pafolacianine)
BLZ-100^41–43^	Highlights brain and CNS solid state tumours.	Binds to chloride ion channels overexpressed in tumours.	Cyclic peptide derived from chlorotoxin coupled to ICG (a NIR dye).	In Phase II and III clinical trials for visualisation of brain and CNS tumours.	675/740 nm	30 mg per kg
LUM015^49,51,53^	Labels breast cancer tissue for imaging during surgical resection.	Cathepsins present in higher concentrations in cancer.	Peptide sequence exhibits fluorescence once cleaved by cathepsins.	Phase III clinical trial investigations underway.	649/670 nm	1.0 mg per kg
ICG^54,55,59^	Perfusion based investigations and tumour detection.	ICG has no binding target	Tricarbocyanine dye (available in sulfonated and nonsulfonated form).	Initial FDA approval granted in 1959	784/814 nm	0.1-5.0 mg per kg (dependant on investigation).

## Ethical approval

Ethical approval is not relevant or required for this review.

## Sources of funding

PhD of primary author funded by EPRSC and MRC via the OPTIMA CDT (grant reference EP/L016559/1).

## Author contribution

S.R. – primary author (research collection, writing, and design). P.M.B provided guidance on clinical aspects of review, writing, and design. Dr A.L. and Prof M.B.: paper design and writing.

## Conflicts of interest disclosure

No known conflicts of interest.

## Research registration unique identifying number (UIN)

1. Name of the registry: Not applicable.
2. Unique Identifying number or registration ID: Not applicable.
3. Hyperlink to your specific registration (must be publicly accessible and will be checked): Not applicable.

## Guarantor

Professor Mark Bradley.

## Data availability statement

This review article contains no original data. All data reviewed throughout this article can be found in the referenced articles or via the clinical trial identifier that are also referenced throughout.
